# Steamboat Excursion

**Published:** 1867-06

**Authors:** 


STEAMBOAT EXCURSION.

At 12 o'clock the Louisville mail steamer America presented a lively appearance. It will be remembered that she is the magnificent two-storied steamboat, built expressly for the Louisville line, her equal is hardly to be found on any waters east or west. Tickets of invitation by the City Council had been distributed liberally among official and prominent men in the city, who, with their ladies, to the number of six or eight hundred, stood on the decks and balconies to welcome the city's guests. The members of the Convention, with their ladies, arriving about half-past 12, and mingling with our people in the gay cabins, or in the genial sunshine on deck, presented the liveliest throng we have seen for many a day. A band of music entertained the company, if such a thing were needed in such a concourse of intelligent and socially disposed people. The guests cared more for taking a view of the city from the upper decks, as the noble vessel glided down its river shore, and further down to admire the hill-sides, covered with vineyards, and the valleys filled with gardens. At 1 o'clock the band took position in the middle balcony of the cabin, and to a lively march the company assembled around the extensive tables of the steamer, and partook of a grateful lunch.

Dr. G. H. Dougherty, Chairman of the Council Committee on Excursion, made a brief address of welcome to the visitors and assured them of the city's pleasure in having them among us.

The trip was extended only to North Bend and return, without landing, as the vessel had to be back in time to receive her cargo and passengers for departure to Louisville at 5 o'clock. At 3 o'clock the company left the boat, greatly pleased with the brief season of recreation.

### FOURTH DAY'S PROCEEDINGS.

The Association met this morning, the fourth day of its session, with about half the usual attendance, President Askew in the chair.

The following named gentlemen were elected members by invitation:—Drs. John Dillard, of Lexington, Ky.; A. J. Larey, Mt. Pleasant, Kansas; S. S. Gray and A. S. Ashton, Piqua, O.; John A. Warder and J. D. Stubbles, Cincinnati, O.; Dr. Marsden, of Quebec, Canada; G. M. Kellogg, of Iowa; Dr. Maloy, of Cincinnati.

